# Corticosteroid Versus Hyaluronic Acid Intra-articular Injections for Pain Relief in Trapeziometacarpal Joint Osteoarthritis: A Systematic Review and Meta-Analysis

**DOI:** 10.7759/cureus.96855

**Published:** 2025-11-14

**Authors:** Roya Khorram, Nikita Golovachev, Mohammad Poursalehian, Pedro Beredjiklian, Amir R Kachooei

**Affiliations:** 1 Orthopaedics, Rothman Orthopaedics Florida at AdventHealth, Orlando, USA; 2 Medical Student, Joint Reconstruction Research Center, Tehran University of Medical Sciences, Tehran, IRN; 3 Orthopaedics, Division of Hand Surgery, Rothman Orthopaedic Institute, Philadelphia, USA; 4 Orthopaedics, University of Central Florida, Orlando, USA; 5 Orthopaedics, Orthopedics Research Center, Mashhad University of Medical Sciences, Mashhad, IRN

**Keywords:** carpometacarpal joint, corticosteroid, hyaline, injection, pain, trapeziometacarpal joint osteoarthritis

## Abstract

Corticosteroid (CSI) and hyaluronic acid injections (HAI) are common non-operative treatments for trapeziometacarpal joint osteoarthritis (TMJO), but their comparative long-term efficacy is debated. This systematic review and meta-analysis aimed to compare pain relief between intra-articular CSI and HAI in patients with TMJO at six-month follow-up. We hypothesized there would be no significant difference between the treatments at six months. Following PRISMA guidelines, a systematic search was conducted in PubMed, Cochrane, Embase, and Medline databases. We included only randomized controlled trials (RCTs) comparing CSI versus HAI for TMJO that reported visual analog scale (VAS) scores with a minimum follow-up of six months. Four RCTs met the inclusion criteria with a total of 222 patients, 48% (n=107) of whom were treated with corticosteroid injection (CSI) and 52% (n=115) were treated with HAI between 2005 and 2015, with a follow-up period of 6 months. At 1-month follow-up, CSIs provided significantly greater pain relief than HAIs (mean difference (MD) of 0.73 (95% CI: 0.02 to 1.43; P = 0.043; I² = 0%). At 3 months, no significant difference in VAS pain scores was observed between the two groups (MD = 0.14, 95% CI: -0.49 to 0.76; P = 0.670; I² = 42%). The results remained nonsignificant (MD = 0.21 (95% CI: -0.70 to 1.11; P = 0.654; I² = 0%) at 6-month follow-up. CSI offers a pain relief advantage over HAI in TMJO treatment at the one-month follow-up period, but this benefit is not sustained over time.

## Introduction and background

Trapeziometacarpal joint osteoarthritis (TMJO) is a prevalent degenerative condition, particularly affecting postmenopausal women [1]. The prevalence of TMJO varies across populations, with distinct patterns of joint involvement potentially influencing treatment response, further complicating therapeutic decision-making [2]. TMJO manifests as pain, stiffness, and impaired hand function, significantly impacting daily activities and quality of life [3,4].

The condition can be treated through conservative and surgical methods [5-9]. Non-operative treatments, including intra-articular injections, are commonly employed to alleviate symptoms, with corticosteroids and hyaluronic acid (HA) derivatives being two widely studied and available options [10]. Corticosteroid injections (CSIs) have demonstrated short-term pain relief and functional improvement in TMJO, though their long-term efficacy remains debated [3]. In contrast, hyaluronic acid injections (HAI), including high-molecular-weight derivatives like Hylan G-F 20, aim to restore synovial fluid viscosity and have shown significant pain reduction and functional improvement at six months in patients with bilateral TMJO [11]. However, comparative studies suggest variable outcomes, with some indicating that CSI provides more sustained pain relief and functional benefits compared to HAI at longer follow-ups, while others report comparable efficacy [1,10,12].

Given the conflicting evidence and lack of consensus on the optimal intra-articular therapy, this systematic review and meta-analysis aims to compare the efficacy of corticosteroid versus HAIs for pain relief in TMJO at six months follow-up, synthesizing data from randomized controlled trials to guide clinical practice. We hypothesized no significant difference in pain at 6 months between CSI and HAI in patients with TMJO.

## Review

Methods

This systematic review and meta-analysis was conducted following the Preferred Reporting Items for Systematic Reviews and Meta-Analyses (PRISMA) guidelines [13].

Search Strategy

Two researchers (R.K. and N.G.) systematically searched PubMed, Embase, Cochrane Library, and Medline from 2005 until September 23rd, 2025. Moreover, 50 pages of Google Scholar were manually queried to include potentially relevant studies. The references of identified studies were manually screened for relevant studies. Grey literature was not checked in this study, as grey literature often lacks rigorous peer review, is frequently incomplete or difficult to access in a reproducible manner, and may introduce bias due to limited methodological transparency. The search strategy incorporated a combination of MeSH terms and combined truncated key terms were used to construct search strategy queries as follows: ((thumb basal joint) OR (cmc1) OR (first carpometacarpal)) AND ((arthritis) OR (osteoarthritis) OR (OA)) AND ((NSAID) OR (analgesics) OR (medication) OR (hyaluronic) OR (hyaluronidate) OR (hylan) OR (corticosteroid) OR (steroid) OR (csi) OR (corticosteroid injection)). The systematic search strategy was designed according to the PICO (Patient-intervention-Comparison-Outcome) framework to assess the pain relief in TMJO following intra-articular CSI compared with HAI at 6 months follow-up.

Screening and Eligibility Criteria

Two authors (R.K. and N.G.) independently screened titles and abstracts. Duplicates, non-English papers, case reports, and biomechanical/cadaveric studies were excluded. The full texts of the potentially eligible articles were then screened, considering the inclusion and exclusion criteria. Randomized controlled trials (RCTs) that examined visual analog scale (VAS) scores pre- and post-CSI compared to HAI for TMJO were considered. The exclusion criteria were 1) non-English studies, 2) studies involving surgical procedures or postoperative care, 3) case reports, 4) review articles, and 5) studies that did not incorporate VAS for pain assessment. The classification of evidence followed the Oxford Centre for Evidence-Based Medicine guidelines, with a specific focus on RCTs categorized as level I evidence.

Data Extraction

**Figure 1:**
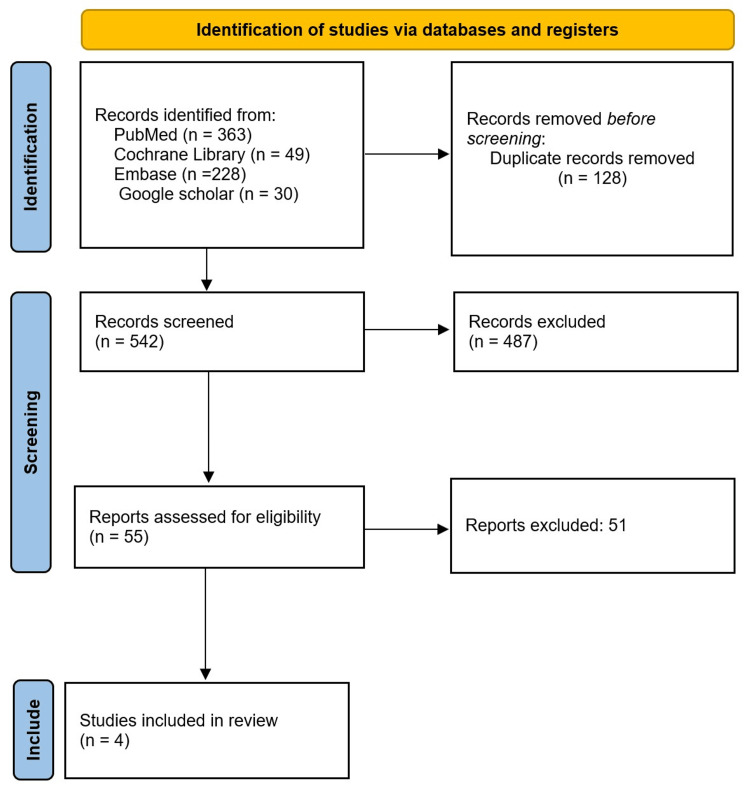
PRISMA flow diagram of the assessed studies. PRISMA: Preferred Reporting Items for Systematic Reviews and Meta-Analyses

Two independent reviewers (N.G. and R.K.) extracted data from the full texts of the included studies using a pre-designed Excel sheet on study design, sample characteristics, intervention details, and outcome measures. Disagreements were resolved through discussion or consultation with the corresponding author. The PRISMA flowchart in Figure 1 shows the screening process.

Methodological Quality Assessment

Two investigators (R.K. and M.P.) independently assessed the quality of each included study. The Risk of Bias Assessment Tool (RoB; The Cochrane Collaboration, London, UK) was utilized to evaluate potential sources of bias in RCTs, considering multiple domains such as randomization, allocation concealment, blinding, incomplete outcome data, and selective reporting [14].

Statistical Analysis

Meta-analysis was performed only for studies comparing HAI versus CSI, as this was the only intervention with a sufficient number of comparable randomized controlled trials. The primary outcome was pain reduction, measured by the visual analog scale (VAS) at 1-, 3-, and 6-month follow-up intervals. Data were extracted as mean changes from baseline to each follow-up time point (1, 3, and 6 months) for the CSI and HAI groups, along with associated standard deviations (SDs) or standard errors (SEs). For the one study that reported outcomes as mean ± SE instead of SD, SE was converted to SD using the formula SD = SE × √n, where n represents the sample size per group at the respective time point. There were no instances of missing SDs or medians, as all included studies provided complete variability measures for the VAS outcome. Publication bias for the HAI versus CSI comparison was assessed using Egger’s test.

For each time point, we calculated the mean difference (MD) in VAS scores between treatment groups. A fixed-effect model was applied when statistical heterogeneity was low (I² < 50%), while a random-effects model was used when heterogeneity was substantial (I² ≥ 50%). Heterogeneity was assessed using the I² statistic and the Chi-square test. All statistical analyses were performed using Comprehensive Meta-Analysis software (CMA, version 3.1; Biostat Inc., Englewood, USA).

Results

Study Selection and Characteristics

An initial search yielded 670 records. After removal of duplicates, 542 studies remained, of which 487 were excluded by screening the title and abstract. Based on the full-text review, four studies were included in the current study. A total of 222 patients were analyzed across the studies, with sample sizes ranging from 40 to 88 participants. The studies predominantly included female patients (199 out of 222), with a female ratio ranging from 80% to 100%. The mean age of participants ranged from 61 to 65 years. Two studies [1,15] reported disease severity using the Eaton and Littler classification (stage II), one study [12] used Kellgren-Lawrence grading (grades I-III), and one study [10] did not exclude patients based on radiographic grading. The HA dosage across studies ranged from 15 mg to 16 mg per injection, with one study [10] explicitly using Hylan G-F 20, while the specific HA formulations in the other studies were not detailed but referred to as hyaluronate. The CSIs varied in composition, including betamethasone [10,12], triamcinolone acetonide [1], and methylprednisolone acetate [15], with equivalent doses ranging from 16 to 48 mg (Table 1).

**Table 1:** Description of articles and study demographics in the analyzed studies. F: Female; M: Male; CS: Corticosteroid; HA: Hyaluronic Acid Heyworth et al. [10] included a placebo group (n=18) not analyzed in this meta-analysis.

Author, Year	Patients	F ratio	F/M	Age	HA dosage	Name of CS	Equivalent dose	Stage of disease
Monfort et al., 2015 [12]	88	87.70%	77/11	Mean 62.9	15mg	Sodium betamethasone sodium phosphate-betamethasone acetate	48 mg	Kellgren-Lawrence grade I-III
Bahadir et al., 2009 [1]	40	100%	HA: 20	Hyaluronate: mean 60.8	15mg	Triamcinolone acetonide	20 mg	Eaton stage II
			CS: 20	Steroid: mean 62.9				
Heyworth et al., 2008 [10]	42	Hylan: 80%	HA: 16/4	Hylan: mean 64	16mg	Sodium betamethasone sodium phosphate-betamethasone acetate	16 mg	Patients were not excluded based on radiographic grade
		Steroid: 91%	CS: 20/2	Steroid: mean 65				
		Control: 89%	Control: 16/2	Control: mean 64				
Stahl et al., 2005 [15]	52	Steroid: 84%	CS: 21/4	Steroid: mean 62	15mg	Methylprednisolone acetate	40 mg	Eaton stage II
		Hyaluronate: 92.3%	HA: 25/2	Hyaluronate: mean 62.6				

Quality Assessment

The quality assessment using the Revised Cochrane Risk-of-Bias Tool (RoB) showed that one study [10] had a low overall risk of bias, while the others [1,12,15] presented some concerns, primarily in the randomization process and selection of reported results (Table 2).

**Table 2:** Quality assessment of the randomized controlled trials (RCT) using the Revised Cochrane Risk-of-bias tool for randomized trials (RoB) D1: Randomization Process; D2: Deviations from Intended Interventions; D3: Missing Outcome Data; D4: Measurement of Outcome D5: Selection of Reported Result

Study ID	D1	D2	D3	D4	D5	Overall Risk
Bahadir et al., 2009 [1]	Low risk	Some concerns	Low risk	Low risk	Some concerns	Some concerns
Heyworth et al., 2008 [10]	Low risk	Low risk	Low risk	Low risk	Low risk	Low risk
Monfort et al., 2015 [12]	Some concerns	Low risk	Low risk	Low risk	Some concerns	Some concerns
Stahl et al., 2005 [15]	Some concerns	Some concerns	Low risk	Low risk	Some concerns	Some concerns

One-Month Follow-Up

**Figure 2:**
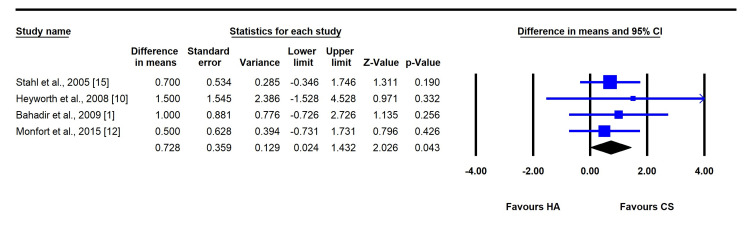
Forest plot comparing the mean difference between corticosteroid (CS) and hyaluronic acid (HA) injections (at one-month follow-up).

At one month, CSI demonstrated significantly greater pain relief compared to HAI, with a mean difference (MD) of 0.73 (95% CI: 0.02 to 1.43; P = 0.043; I² = 0%) favoring CSI. Both the CSI and HAI groups showed significant reductions in VAS scores from baseline values. No publication bias was detected (P > 0.05) (Figure 2).

Three-Month Follow-Up

**Figure 3:**
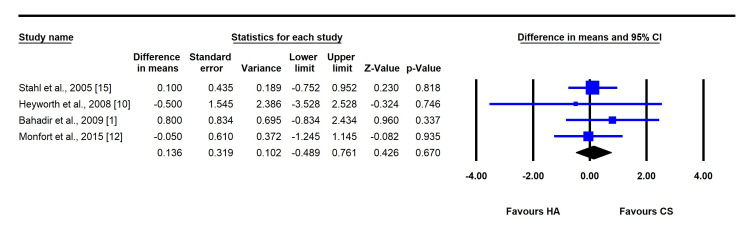
Forest plot comparing the mean difference between corticosteroid (CS) and hyaluronic acid (HA) injections (at three-month follow-up).

At three months, there was no statistically significant difference in VAS pain scores between the two groups (MD = 0.14, 95% CI: -0.49 to 0.76; P = 0.670; I² = 42%). Both groups maintained improvements compared with baseline, although no between-group difference was observed. Again, publication bias was not evident (P > 0.05) (Figure 3).

Six-Month Follow-Up

**Figure 4:**
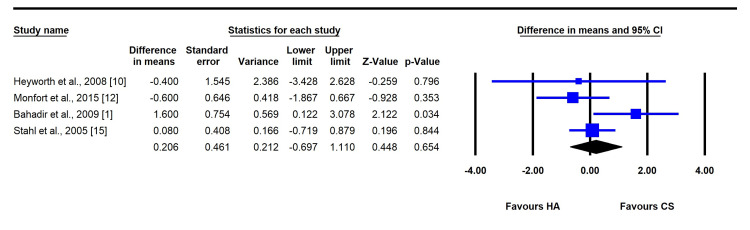
Forest plot comparing the mean difference between corticosteroid (CS) and hyaluronic acid (HA) injections (at six-month follow-up).

At six months, the comparison remained nonsignificant, with a mean difference of 0.21 (95% CI: -0.70 to 1.11; P = 0.654; I² = 0%). Both the CSI and HAI groups continued to show sustained reductions in VAS scores from baseline, without a significant difference between treatments. No publication bias was detected (P > 0.05) (Figure 4).

Discussion

This meta-analysis investigated the comparative effectiveness of CSI and HAIs for pain relief in patients with TMJO across three time points. The primary finding of this study was that CSIs resulted in superior short-term pain reduction at one month compared to HAIs, while no significant differences were observed at three and six months. These results suggest that despite an immediate pain relief with CSI, there is a rapid decline in its efficacy over time. Moreover, HAI shows a minimal but sustained pain relief. Also, across all included trials, both groups experienced significant improvements from baseline, confirming that both corticosteroid and hyaluronic acid injections are effective for short- to mid-term symptomatic relief.

The findings of this study are consistent with the rapid anti-inflammatory effects of CSIs reported in prior studies [16]. In a study by Bahadir et al. [1], 40 patients with TMJO received either a single injection of triamcinolone acetonide or a series of three weekly injections of sodium hyaluronate. Their findings showed statistically significant higher improvement in VAS score in the corticosteroid group compared to HA at one-month post-injection. Heyworth et al. [10] conducted a double-blinded randomized trial involving 60 patients, comparing corticosteroid, HA, and placebo injections. They found that CSIs were associated with the most pronounced pain relief among the study groups at one-month follow-up, although the difference was not statistically significant. These consistencies suggest that CSI may act more rapidly due to its anti-inflammatory properties [11]. In contrast, Trellu et al. [17] found no statistically significant difference in pain outcomes between HA and corticosteroids at early follow-up points (week 4 or 12), suggesting equivalent short-term efficacy. This discrepancy may arise from differences in injection protocol (e.g., single vs. multiple injections), stage of the arthritis, study quality, and sample size heterogeneity.

At three months, our meta-analysis revealed no statistically significant difference in VAS pain scores between patients treated with HAI and those receiving CSI. This finding aligns with Monfort et al. [12], who conducted a single-blinded RCT involving 88 patients with TMJO. Participants were randomized to receive either three weekly injections of betamethasone or sodium hyaluronate. At the 12-week follow-up, both groups demonstrated significant pain reduction, with no meaningful difference between treatments. The findings of Trellu et al. [17] further support this mid-term equivalence.

A study by Trellu et al. [17] examined intra-articular injections for thumb osteoarthritis, incorporating trials comparing HAI and CSI to placebo, as well as indirect comparisons between CSI and HAI. In contrast to their broader analysis, which included comparisons of HAI, CSI, and placebo and evaluated functional outcomes (e.g., function, pinch strength), our meta-analysis focuses solely on direct comparisons of CSI and HAI for pain relief. Our study employed stricter inclusion criteria, including only randomized controlled trials (RCTs) with VAS pain scores reported for at least 6 months and excluding studies with placebo groups or inadequate follow-up. The resulting four studies are more homogenous and offer a more precise evaluation of the long-term comparative effectiveness of CSI and HAI, addressing the ongoing clinical question of which treatment provides better pain control in thumb basal joint osteoarthritis. Their findings indicated that neither treatment showed superiority over the placebo at the 3-month follow-up, suggesting that neither CSI nor HAI acts as a disease modifier or alters the progression of arthritis [18]. CSIs and HAIs are best classified as symptom-modifying rather than disease-modifying therapies [19-22]. Although hyaluronic acid (HA) has shown some biologic potential for chondroprotection in preclinical studies, clinical evidence remains heterogeneous and has not demonstrated reproducible structural modification of OA progression [20,21]. Similarly, long-term corticosteroid use provides symptomatic benefits without altering joint space loss or disease trajectory [18,23].

Bahadir et al. [1] reported a continued improvement in pain score, favoring corticosteroids over HA at the three-month follow-up. However, the difference between groups was not statistically significant. Their study found consistently better outcomes in the corticosteroid group, although the effect declined over time.

At the six-month follow-up, our meta-analysis demonstrated no statistically significant difference in pain improvement between HAI and CSI. This result is supported by Stahl et al. [15], who found that although corticosteroids offered faster initial pain relief, no significant difference in pain was observed between groups at six months. Monfort et al. [12] similarly reported no significant difference in VAS pain scores between CSI and HAI groups at 24 weeks. Conversely, Bahadir et al. [1] reported continued superiority of corticosteroids at six months, significantly reducing VAS pain scores. However, the effect was fading for both CSI and HAI over time. This divergence may reflect differences in disease severity, injection protocol (single versus multiple injections), or patient selection criteria, as their cohort may have included individuals with more advanced joint degeneration.

Limitations

Several limitations should be acknowledged in this meta-analysis. First, the number of included studies was relatively small, and most studies had modest sample sizes, which may limit the generalizability of the findings. Second, heterogeneity in study design, injection protocols, such as type and dose of corticosteroid or hyaluronic acid, single versus multiple injections, and disease severity grading could have influenced the pooled outcomes. Third, outcome reporting was largely restricted to pain scores using the visual analog scale; functional outcomes such as grip strength, pinch strength, and quality of life measures were inconsistently reported and therefore could not be quantitatively synthesized. Fourth, follow-up was limited to six months, preventing assessment of longer-term effects beyond this period.

## Conclusions

This meta-analysis found that across all follow-up intervals, both CSIs and HAIs demonstrated significant improvements in pain compared with baseline, confirming that each offers symptomatic benefit. Also, CSIs provide superior short-term pain relief in patients with TMJO when compared to HAIs at one-month follow-up. However, this initial advantage diminishes over time, with our results showing no statistically significant difference in pain scores between the two treatments at either three- or six-month follow-ups. Therefore, for patients seeking rapid, short-term relief, a corticosteroid injection is the more effective option. For sustained management, HAI provides a similar level of pain relief as CSIs at three and six months. Clinicians should counsel patients that while both injections can alleviate symptoms, neither has been shown to alter the natural course of the underlying arthritis.
